# Fitness Restoration of a Genetically Tractable Enterococcus faecalis V583 Derivative To Study Decoration-Related Phenotypes of the Enterococcal Polysaccharide Antigen

**DOI:** 10.1128/mSphere.00310-19

**Published:** 2019-07-10

**Authors:** Sylviane Furlan, Renata C. Matos, Sean P. Kennedy, Benoît Doublet, Pascale Serror, Lionel Rigottier-Gois

**Affiliations:** a UMR Micalis, INRA, AgroParisTech, Université Paris-Saclay, UMR1319, Jouy-en-Josas, France; b Center of Bioinformatics, Biostatistics and Integrative Biology (C3BI), Institut Pasteur, USR 3756 CNRS, 75015, Paris, France; c UMR ISP, INRA, Université Tours, UMR 1282, Nouzilly, France; University of Iowa

**Keywords:** *Enterococcus faecalis*, *epa* variable region, genome and SNP analyses, growth fitness, host adaptation, platform strain

## Abstract

Commensal and generally harmless in healthy individuals, Enterococcus faecalis causes opportunistic infections in immunocompromised patients. Plasmid-cured E. faecalis strain VE14089, derived from sequenced reference strain V583, is widely used for functional studies due to its improved genetic amenability. Although strain VE14089 has no major DNA rearrangements, with the exception of an ∼20-kb integrated region of pTEF1 plasmid, the strain presented significant growth differences from the V583 reference strain of our collection (renamed VE14002). In the present study, genome sequencing of strain VE14089 identified additional point mutations. Excision of the integrated pTEF1 plasmid region and sequential restoration of wild-type alleles showing nonsilent mutations were performed to obtain the VE18379 reference-derivative strain. Recovery of the growth ability of the restored VE18379 strain at a level similar to that seen with the reference strain points to GreA and Spx as bacterial fitness determinants. Virulence potential in Galleria mellonella and intestinal colonization in mouse demonstrated host adaptation of the VE18379 strain equivalent to VE14002 host adaptation. We further demonstrated that deletion of the 16.8-kb variable region of the *epa* locus recapitulates the key role of Epa decoration in host adaptation, providing a genetic system to study the role of specific *epa*-variable regions in host adaptation independently of other genetic variations.

**IMPORTANCE** E. faecalis strain VE14089 was derived from V583 cured of its plasmids. Although VE14089 had no major DNA rearrangements, it presented significant growth and host adaptation differences from the reference strain V583 of our collection. To construct a strain with better fitness, we sequenced the genome of VE14089, identified single nucleotide polymorphisms (SNPs), and repaired the genes that could account for these changes. Using this reference-derivative strain, we provide a novel genetic system to understand the role of the variable region of *epa* in the enterococcal lifestyle.

## Abstract (precis)

E. faecalis strain VE14089 was derived from V583 cured of its plasmids. Although VE14089 had no major DNA rearrangements, it presented significant growth and host adaptation differences from the reference strain V583 of our collection. To construct a strain with better fitness, we sequenced the genome of VE14089, identified single nucleotide polymorphisms (SNPs), and repaired the genes that could account for these changes. Using this reference-derivative strain, we provide a novel genetic system to understand the role of the variable region of *epa* in the enterococcal lifestyle.

## INTRODUCTION

The gastrointestinal tract harbors a complex microbial community that is beneficial to its host but that also represents a reservoir of opportunistic pathogens or pathobionts in case of dysbiosis. Among the pathobionts in humans, Enterococcus faecalis is a pioneer Gram-positive species of the infant gastrointestinal tract that persists in adults as part of the subdominant species of the core microbiota (1). As with other opportunistic pathogens, some isolates have the potential to cause severe infections in hospitalized or fragile patients (2) or in those with predisposing conditions, such as reduced immune function (3, 4). Data from clinical studies indicate that intestinal colonization precedes systemic infection and that antimicrobial therapies increase the risk of colonization with vancomycin-resistant enterococci (5). More than 70 virulence factors have been identified, with different models used to study enterococcal pathogenesis (reviewed in references 6 and 7). Cell wall glycopolymers, including the ubiquitous rhamnopolysaccharide enterococcal polysaccharide antigen (Epa), have been shown to play a major role in enterococcal pathogenesis. Epa is implicated in adhesion to intestinal mucus and in translocation into epithelial cells, resistance to phagocytosis, colonization and virulence in animal models, and antibiotic resistance (8–13). Colonizing or invasive enterococcal isolates from hospitalized patients are commonly resistant to antibiotics in general and to vancomycin treatment in particular. Indeed, most hospital isolates belong to specific high-risk enterococcal clonal complexes (HIRECC) of E. faecalis (14, 15). Isolates of HIRECC-2 (clonal complex 2 [CC2]) are among the most common causes of E. faecalis infections in the United States and in several European countries (16, 17). Strain V583 belongs to CC2 and was the first vancomycin-resistant enterococcus (VRE) isolate reported in the United States (18). Since the report by Hancock and Perego was published (19), several research groups have attempted to genetically modify E. faecalis reference strain V583. Due to the presence of at least two plasmids (20), genetic manipulation of E. faecalis strain V583 remains challenging (21). To avoid problems related to the presence of replicating plasmids and to simplify genetic modification in this strain, we used a plasmid-cured derivative of strain V583 named VE14089 that was obtained after novobiocin and thermic shocks (22). Strain VE14089 was generated from our clone of the V583 strain that was renamed VE14002 and that lacks the pTEF3 plasmid (23). The VE14089 strain has previously shown a growth defect in comparison to the VE14002 parental strain; the defect was more pronounced under aerobic than anaerobic conditions (22). However, with the exception of a 20.5-kb region of pTEF1 (from *efa0063* up to *efa0006*) integrated between chromosomal genes *ef3209* and *ef3210*, the VE14089 strain had not undergone any other major DNA rearrangements (22). Although a large dissemination of VE14089 in specialized research laboratories has been reported (see, e.g., 24–32), it remains characterized only partially and its genome sequence and comparison to V583 have not yet been described.

Here we report the construction of the genetically tractable E. faecalis strain VE18379 representative of the enterococcal clonal complex CC2 that shows restored fitness to permit functional studies. First, modifications potentially affecting fitness of strain VE14089 were identified by whole-genome sequencing. Then, after deletion of the 20.5-kb region of pTEF1, nonsilent mutations were sequentially restored to obtain the E. faecalis VE18379 strain. Genome resequencing and in-depth phenotypic characterization confirmed that strain VE18379 growth fitness and host adaptation were restored to a level equivalent to that of the reference E. faecalis strain VE14002. Finally, as a first proof of the genetic tractability of E. faecalis strain VE18379, deletion and complementation of the 16.8-kb *epa* variable (*epa*_var_) region corroborated the role of this variable region in host adaptation and resistance to antibiotics.

## RESULTS

### Identification of mutations in E. faecalis strain VE14089 induced by plasmid curing.

**FIG 1:**
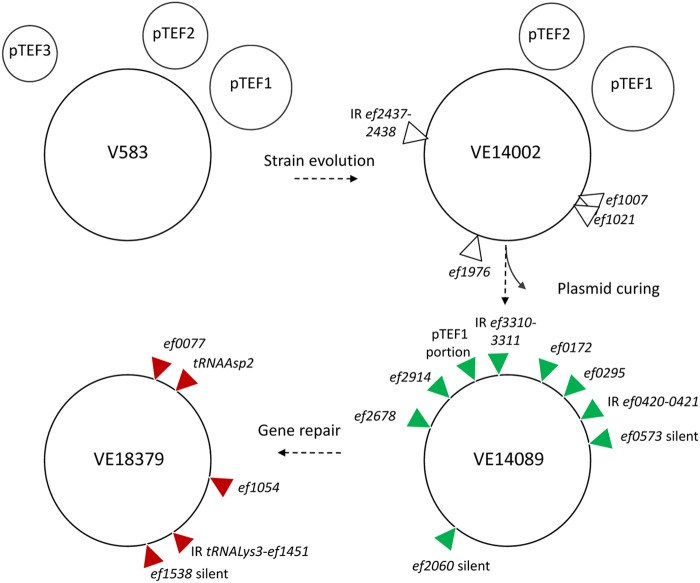
Genome comparison between E. faecalis strains V583, VE14002, VE14089, and VE18379. SNPs appears in white for VE14002 compared to V583, in green for VE14089 compared to VE14002, and in red for VE18379 compared to VE14089. Accession numbers are AE016830, CP039296, and CP039548 for V583, VE14089, and VE18379, respectively. Variants identified between VE14089 and V583 were confirmed by PCR using VE14089 DNA and VE14002 DNA as the templates. IR, inverted repeat.

The genome of the plasmid-cured E. faecalis VE14089 strain was sequenced and compared to the genome of the reference V583 strain (33). In addition to the 20.5-kb insertion between genes *ef3209* and *ef3210*, 46 differences, including deletions, insertions, and single nucleotide polymorphism (SNPs), were detected between the VE14089 and V583 genome sequences. Of these, 34 differences were reported previously by Palmer et al. (34), leading us to consider them to be real differences which may be due to sequencing errors of the initial V583 genome and/or may reflect the evolution of independent isolates of the original V583 strain (see Table S1 in the supplemental material). The other 12 differences were confirmed after PCR amplification and sequencing of the corresponding regions of VE14089 and VE14002 (strain V583 of our collection [devoid of pTEF3]) (Table 1). Nine differences are located within open reading frames (ORFs), and four are found in intergenic regions. One deletion and three missense SNPs are found in both strains VE14002 and VE14089, being specific to our laboratory strains. The 5-bp deletion in *ef1007* generates a predicted protein of 243 residues annotated as a regulator of sugar fermentation. Noticeably, *ef1007* is annotated as mutated in the V583 genome due to a frameshift mutation that corresponds to the 5 bp missing in our strains. This insertion is also absent in resequenced V583 genomes found in GenBank (accession numbers AHYN01000020.1 and ASWP01000005.1) and in all available E. faecalis genomes, suggesting an assembly error in the reference genome. The remaining eight SNPs were specific to VE14089 and are likely due to the plasmid curing treatment (Table 1; see also Fig. 1). The single nonsense SNP was found in gene *ef0295*, predicted to encode subunit J of a V-type ATP synthase. This protein acts as a primary ion pump transporting Na^+^ or K^+^ ions. The three other substitutions predicted as nonneutral are in annotated transcription factors. The substitution in the DNA-binding domain of the predicted sugar-binding transcriptional regulator EF0172 may affect efficient regulation of its unknown targets. E. faecalis Spx regulator EF2678 is a global transcriptional regulator, which modulates RNA polymerase specificity in response to oxidative stress under aerobic conditions. It promotes colonization of the peritoneum and dissemination in the blood in mice (35). Transcription elongation factor GreA acts on the fidelity and processivity of RNA polymerase (36). However, the effect of the substitutions on these two regulators was difficult to predict. Finally, two SNPs are predicted as silent in *ef0573* and *ef2060*. Overall, the plasmid-cured VE14089 strain lacks pTEF2 and pTEF1 (with the exception of the 20.5-kb region integrated between *ef3209* and *ef3210*) and harbors 6 point mutations in ORFs relative to our VE14002 strain, conferring 1 truncated gene (*ef0295*) and 3 missense mutated ORFs (in *ef0172*, *ef2678*, and *ef2914*) (Fig. 1).

**TABLE 1:** Variations in E. faecalis strain VE14002 and plasmid-cured derivative E. faecalis strain VE14089 compared to the reference sequence of E. faecalis V583^*a*^

Reference position^*b*^	Variation type	Variation	Annotation	Predicted function	Amino acid change	Side chain polarity and charge change	SNAP prediction^*c*^
Differences in VE14002 and VE14089							
966197	Deletion	CATGT → −	EF1007	Sugar fermentation stimulation protein	Frameshift	NA	NA
979343	SNP	G → A	EF1021	N-Acetyltransferase	V156I	NA	Neutral
1914901	SNP	G → A	EF1976 (*prmA*)	Ribosomal protein L11 methyltransferase	L74F	NA	Neutral
2357407	SNP	T → C	IR *ef2437–2438*	NA	NA	NA	NA

Differences specific to VE14089							
169833	SNP	C → A	EF0172	Sugar-binding transcriptional regulator, LacI family	T34N	NA	Nonneutral
280276	SNP	C → T	EF0295	V-type ATPase, subunit J	Q45Stop (resulting in protein of 44 aa)	NA	NA
393139	SNP	C → T	IR *ef0420–421*	NA	NA	NA	NA
536621	SNP	C → T	EF0573	Hypothetical protein	Silent	NA	NA
1982291	SNP	G → A	EF2060 (*cydB*)	Cytochrome *d* ubiquinol oxidase, subunit II	Silent	NA	NA
2589844	SNP	T → A	EF2678 (*spx*)	Regulatory protein Spx	N31I	Polar → nonpolar	Nonneutral
2792148	SNP	C → T	EF2914 (*greA*)	Transcription elongation factor	G12E	Nonpolar → acidic polar; neutral → negative	Nonneutral
3080141	Insertion	T → portion pTEF1 (20.5 kb)	IR *ef3209–3210*	NA	NA	NA	NA
3192131 (3212954^*d*^)	SNP	G → T	IR *ef3310–3311*	NA	NA	NA	NA

a aa, amino acid; IR, inverted repeat; NA, not applicable.

b Data indicate positions in E. faecalis V583 reference sequence in NCBI (accession number AE016830).

c SNAP prediction details are available at https://rostlab.org/services/snap2web/. Neutral, no effect on the protein function; Nonneutral, effect on the protein function.

d Position in E. faecalis VE14089, including the pTEF1 portion of 20.5 kb.

10.1128/mSphere.00310-19.6 TABLE S1 Differences detected in VE14002 and VE14089 genomes correlating with those reported previously by Palmer et al. (34). Download Table S1, DOCX file, 0.02 MB. Copyright © 2019 Furlan et al. 2019 Furlan et al. This content is distributed under the terms of the Creative Commons Attribution 4.0 International license.

### E. faecalis strains VE18379 and VE14002 show comparable growth characteristics.

**FIG 2:**
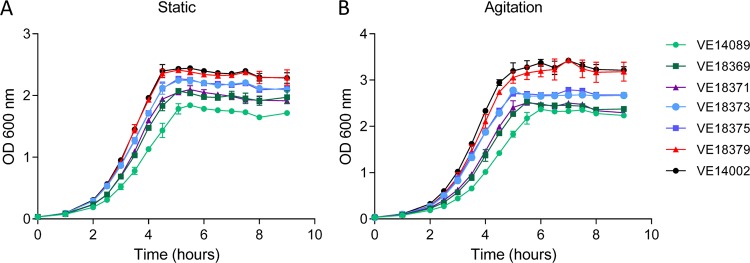
Comparison of growth levels under static conditions (A) and under agitation conditions (B) of E. faecalis strains VE14002 and VE14089 with E. faecalis-derived strains VE18369, VE18371, VE18373, VE18375, and VE18379.

Starting from E. faecalis strain VE14089, a set of isogenic strains was constructed by removing the pTEF1 insertion and by the sequential restoration of wild-type alleles of genes *ef0295*, *ef2914*, *ef0172*, and *ef2678* for which missense and nonsilent SNPs were observed (see details in Fig. 1; see also Fig. S1 in the supplemental material). The growth on solid and in liquid (static and aerated conditions) media of the whole set of strains (VE14089 to VE18379) was compared with the growth characteristics of strain VE14002 (Fig. 2; see also Fig. S1). In solid medium, strains VE18379 and VE14002 showed colonies that were similarly larger than those shown by strain VE14089 (Fig. S1). A subtle but progressive increase in colony size was observed at sequential steps in restoration from VE14089 to VE18379. In liquid medium, the significant growth defect was confirmed for strain VE14089. Generation time and final cell density were progressively restored from strain VE14089 to VE18379 (Table S2). The absence of significant differences in generation time and final cell density between strains VE18379 and VE14002 confirmed the growth fitness recovery of the VE18379 to the level of the reference strain under the tested conditions, with the exception of a slight difference of the generation time under aerated conditions. The removal of a pTEF1-integrated region portion and restoration of the regulatory genes *spx* and *greA* (*ef2914*) strongly affected both the generation time and the cell density. These results indicated that strain VE18379 has growth properties similar to those of reference strain VE14002. Thus, strain VE18379 was considered a reference-derivative strain of enterococcal clonal complex 2 suitable for further genetic manipulations. Gene repair of SNPs and the absence of plasmid insertion were confirmed by genome sequencing of the restored E. faecalis VE18379 strain. Genome sequence comparison of VE18379 and VE14089 uncovered 5 SNPs in VE18379 (Table 2) (Fig. 1). Three SNPs were found to be located within ORFs with no assigned function in E. faecalis (*ef0077*, *ef1054*, and *ef1538*). One gene (*ef1538*) was found to have a synonymous substitution, and the other two (*ef0077* and *ef1054*) showed missense mutations. EF1054 is a predicted ABC transporter permease, and EF1538 is ScpA (segregation and condensation protein A). Sequencing of the intermediary strains from VE14089 to VE18375 revealed the order of appearance of SNPs. SNPs in *ef1538* and *tRNAAsp2* appeared after excision of pTEF1. Mutations in *ef0077* and *ef1054* appeared after repair of *ef0295* and *ef2914* (*greA*), respectively. The last SNP in the intergenic region between *tRNALys3* and *ef1451* appeared in strain E. faecalis VE18379 after repair of *ef2678* (*spx*). In sum, each sequential event of gene repair induced an average of one mutation per cycle but no major DNA rearrangement.

**TABLE 2:** SNPs in E. faecalis strain VE18379 compared to E. faecalis strain VE14089

Reference position^*a*^	Variation type	Variation	Annotation	Predicted function	Amino acid change	Side chain polarity and charge change	SNAP prediction^*b*^
77970	SNP	A → G	EF0077	Conserved hypothetical protein	D110G	Acid polar negative → basic polar	Neutral
255242	SNP	C → A	*tRNAAsp2*	tRNA-Asp2	NA^*c*^	NA	NA
1026912	SNP	C → A	EF1054	ABC transporter, permease protein	W212L	Weak basic nonpolar → nonpolar	Nonneutral
1416209	SNP	G → A	IR *tRNALys3-* *ef1451*	NA	NA	NA	NA
1493077	SNP	C → T	EF1538	ScpA (segregation and condensation protein A)	Silent	NA	NA

a Position in E. faecalis V583 reference sequence in NCBI.

b SNAP prediction details are available at https://rostlab.org/services/snap2web/. Neutral, no effect on the protein function; Nonneutral, effect on the protein function.

c NA, not applicable.

10.1128/mSphere.00310-19.1 FIG S1 Construction of isogenic strains from VE14089 to VE18379 and comparison of colony sizes and of morphology of the isogenic strains with VE14002 (V583). Zoom in corresponds to ×1.4 picture magnification. Download FIG S1, PDF file, 0.2 MB. Copyright © 2019 Furlan et al. 2019 Furlan et al. This content is distributed under the terms of the Creative Commons Attribution 4.0 International license.

10.1128/mSphere.00310-19.7 TABLE S2 Doubling time and cell density of E. faecalis strains derived from VE14089 after sequential repair and comparison with strain VE14002. Download Table S2, DOCX file, 0.02 MB. Copyright © 2019 Furlan et al. 2019 Furlan et al. This content is distributed under the terms of the Creative Commons Attribution 4.0 International license.

### Host adaptation of E. faecalis strain VE18379.

**FIG 3:**
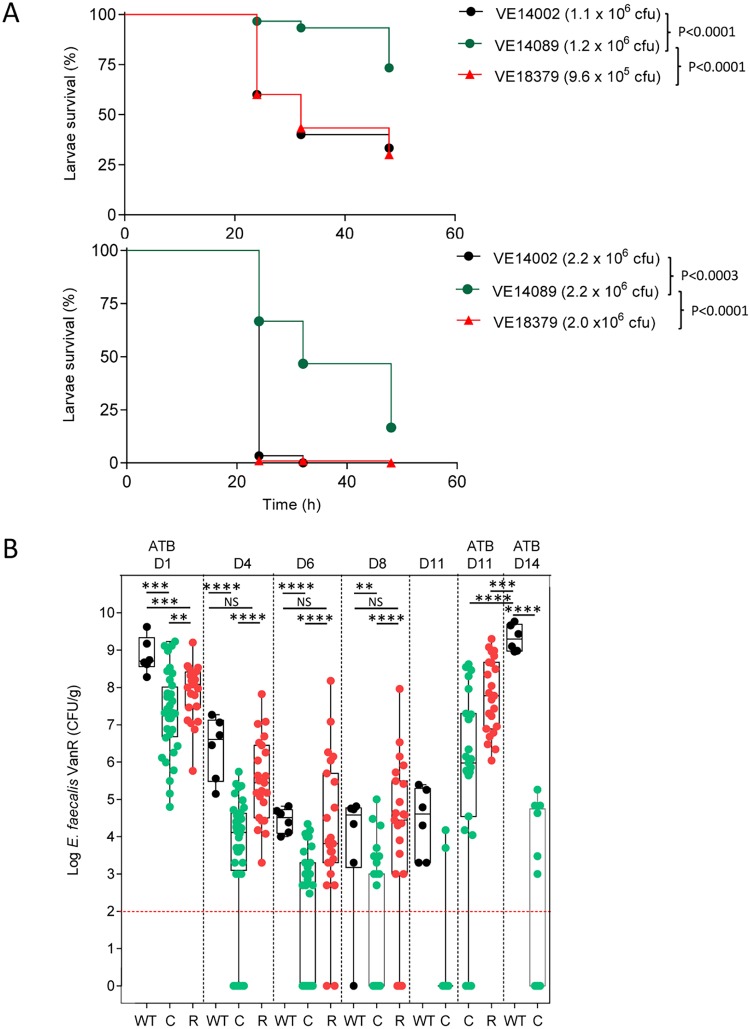
E. faecalis strain VE18379 has similar *in vivo* adaptation to VE14002. (A) Survival of Galleria mellonella after injection with E. faecalis strains VE14002, VE14089, and VE18379 at lower concentrations (top panel) and higher concentrations (bottom panel) (*n* = 30 larvae per group). Survival curves were constructed by the Kaplan-Meier method and compared by log rank analysis. (B) Potential of colonization of E. faecalis strains VE14002 (WT), VE14089 (C), and VE18379 (R) in the gastrointestinal tract of mice after clindamycin treatment and of reemergence after a second clindamycin treatment from D8 to D10 noted ATB D11 for VE14089 (C) and VE18379 (R) (*n* = 23 per group), and from D11 to D13 noted ATB D14 for VE14002 (WT, *n* = 6) and VE14089 (C, *n* = 13). Statistical analysis was based on the Mann-Whitney test.

The reference and derivative strains were examined in *in vivo* assays to assess their ability to resist the host-relevant conditions encountered by the bacteria. The Galleria mellonella insect model (Fig. 3A) was used as a surrogate to evaluate virulence. Strains VE14002 and VE18379 induced comparable levels of lethality for the two doses tested (∼1 × 10^6^ and ∼2 × 10^6^ cells). A dose of 2 × 10^6^ of VE14089 cells was necessary to achieve the same lethality as that seen with strains VE14002 and VE18379 at 1 × 10^6^ cells, demonstrating recovery of the virulence potential of strain VE18379 to a level comparable to that of strain VE14002. To examine colonization abilities in the gut, we performed orogastric inoculation of E. faecalis strains in mice harboring their complex microbiota and imbalanced with clindamycin, a lincosamide targeting the anaerobic bacteria and favoring the proliferation of enterococci (37). To compare the colonization potentials of E. faecalis strains, we performed independent mouse experiments (*n* = 6 to 8 mice for each strain) whose grouped results are presented in Fig. 3B. Similar results representing the behavior of the E. faecalis strains were observed postgavage but with different levels between strains. The levels of carriage of strains VE14002, VE14089, and VE18379 were significantly different at day 1 (D1) after inoculation, with median values of 5.1 × 10^8^, 2.1 × 10^7^, and 1.2 × 10^8^ CFU/g, respectively. For all strains, the carriage decreased after the antibiotic treatment was stopped, corresponding to the resilience of the gut microbiota. No significant differences between the VE14002 and VE18379 strains were observed at D4, D6, and D8. On the same days, significant differences were observed between both strains VE14002 and VE18379 and the cured strain VE14089. The levels of persistence of strains VE14002 and VE18379 after 8 days were also similar (detected in 83% of mice [5/6 and 19/23, respectively]) and were markedly different from that of strain VE14089 (detected in only 30% of mice [11/36]). To assess the ability of strains to reemerge, a second treatment of clindamycin was performed at days 11, 12, and 13 for all mice inoculated with strain VE14002 and for a selection of 13 mice inoculated with strain VE14089. To mitigate the possibility of VE14089 washout, the second treatment of clindamycin was advanced to days 8, 9, and 10 for 23 mice inoculated with VE14089 or with VE18379. The level of VE14002 recovered at day 14 from all mice was equal to the level recovered on day 1. In contrast, VE14089 reappeared at lower levels in 46% of the mice, evincing the lower fitness of the cured strain with respect to reemergence within the gastrointestinal ecosystem. In contrast to strain VE14089 and similarly to strain VE14002, strain VE18379 proliferated in all mice at day 11. However, as for the D1 results, a significant difference in viable counts between VE14002 and VE18379 was observed after the second antibiotic treatment, suggesting a role for plasmids pTEF1 and/or pTEF2 in the initial step of proliferation. These results demonstrate that strain VE18379 provides a V583-like platform that is markedly improved over that represented by VE14089. VE18379 is well-adapted, in terms of colonization persistence, for further functional and basic research studies on host adaptation and intestinal colonization of a representative member of the CC2 clonal complex.

### Deletion of *epa*_var_ region in the reference-derivative E. faecalis strain recapitulates *epa*-associated phenotypes.

**FIG 4:**
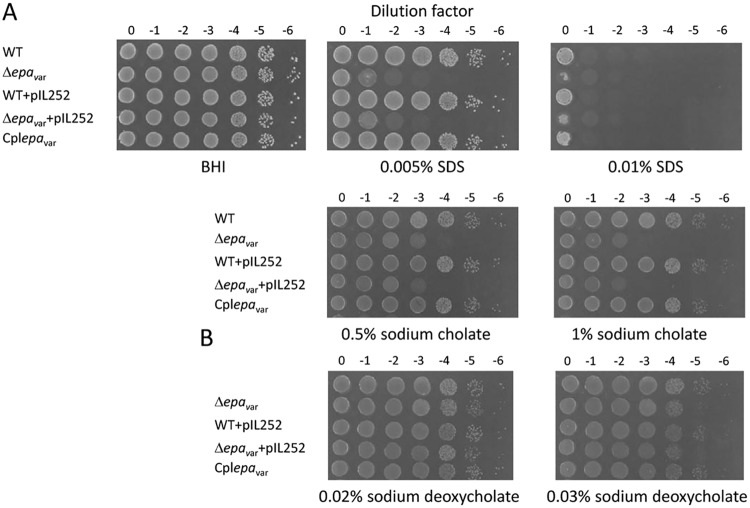
Susceptibility to SDS and to bile salts of Δ*epa* variable region strain and its derivatives. Tenfold serial dilutions were plated on BHI medium or on (i) BHI medium containing 0.005% and 0.01% SDS (A) or on (ii) BHI medium with 0.5% sodium cholate and 1% sodium cholate or (iii) BHI medium with 0.02% sodium deoxycholate and 0.03% sodium deoxycholate (B). WT, VE18379; Δ*epa*_var_, VE18395; WT+pIL252, VE18922; Δ*epa*_var_+pIL252, VE18927; Cpl*epa*_var_, VE18930.

**FIG 5:**
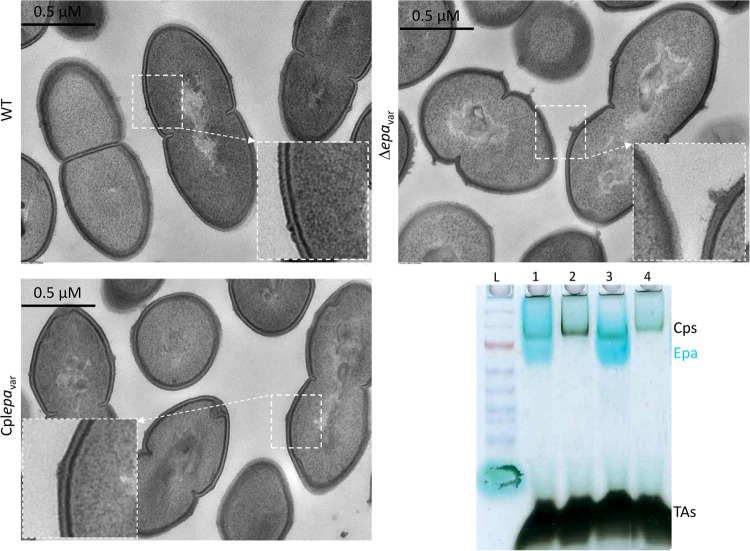
The Δ*epa*_var_ mutant has modified cell wall architecture and polysaccharide profile. Representative transmission electron microscopy micrographs of the wild-type, Δ*epa*_var_ mutant, and complemented strains are presented. The enlargement inside each of the micrographs highlights the presence of the polysaccharide pellicle observed in the wild-type strain (upper left) and the complemented strains (lower left) and its absence in the Δ*epa*_var_ strain (upper right). (Lower right) Blotting was performed for characterization of the polysaccharide profiles of the wild-type plus pIL252 strain (lane 1), Δ*epa*_var_ region plus pIL252 strain (lane 2), complemented Cpl*epa*_var_ strain (lane 3), and Δ*epaX* strain (lane 4). L, ladder; Cps, capsular polysaccharide; Epa, enterococcal polysaccharide antigen; TAs, teichoic acids.

**FIG 6:**
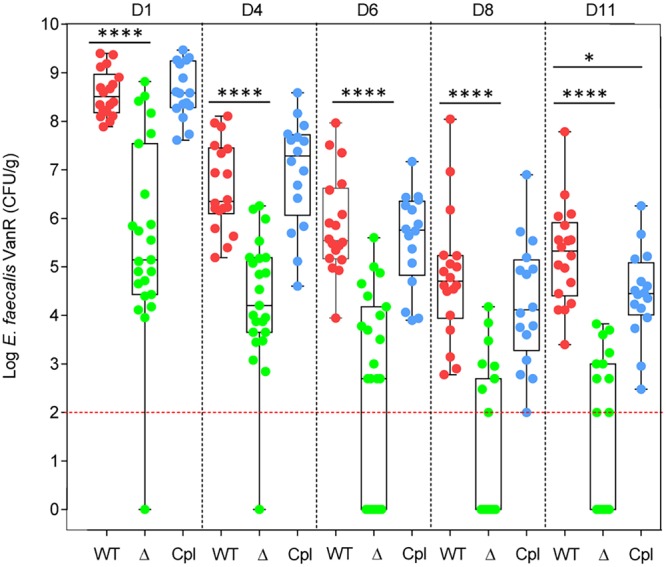
The *epa* variable region is important for intestinal colonization. Data indicate the potential of colonization in the gastrointestinal tract of mice after clindamycin treatment of E. faecalis strain VE18379 (WT, *n* = 18), the Δ*epa*_var_ strain (Δ, *n* = 23), and the complemented *epa*_var_ strain (Cpl, *n* = 16). Statistical analysis was based on the Mann-Whitney test. ****, *P* < 0.0001 (from D1 to D11 for comparisons between the WT and Δ strains); *, *P* = 0.0318 (at D11 for comparisons between the WT and Cpl strains).

The use of isogenic strains to analyze the function of specific Epa decoration is a prerequisite for elimination of other genetic variations. Enterococcal polysaccharide antigen (Epa) biosynthesis is encoded by a locus composed of two gene clusters, one conserved and one presenting genetic variability featuring major differences in Epa between E. faecalis isolates (8, 10, 38). Using the reference-derivative E. faecalis strain VE18379, we constructed E. faecalis VE18395, which is devoid of the variable region of the *epa* locus (Δ*epa*_var_). Genome analysis of VE18395 revealed no SNPs compared to VE18379 and no major rearrangement (accession number CP039549). Growth characterization revealed no significant differences between the wild-type and deletion strains, with the exception of a lower maximum optical density (OD) for the deleted mutant (Fig. S2). The isogenic strains were subjected to a series of assays that examined the *in vitro* ability of the strains to resist detergents on solid medium. The deleted strain had increased sensitivity to SDS, sodium cholate (0.5%), and sodium deoxycholate (0.03%) that could be complemented with a plasmid harboring the 16.8-kb *epa*_var_ region to reach the sensitivity level of the wild-type (WT) strain (Fig. 4). Morphology and cell wall structure in the Δ*epa*_var_, complemented, and wild-type strains were examined using electron microscopy (EM). Strain Δ*epa*_var_ displayed a rounder shape than the wild-type strain and lacked the wild-type strain’s peripheral electron-dense zone, called the pellicle. These morphological characters were recovered in the complemented strain (Fig. 5). Analysis of surface polysaccharides from wild-type and isogenic strains showed that the blue band corresponding to the rhamnopolysaccharide Epa disappeared in the mutant strain and was recovered in the complemented strain (Fig. 5). Antibiotic susceptibility testing of wild-type and Δ*epa*_var_ strains was performed in the presence of 33 different antibiotics by the standard disk diffusion method, and the MIC was determined for 3 selected antibiotics and daptomycin (Table 3). An increase in the susceptibility of the Δ*epa*_var_ strain was observed for vancomycin and for daptomycin and was further confirmed for daptomycin by disk diffusion in the presence of CaCl_2_ (data not shown). In addition, the Δ*epaX* strain, deleted for EpaX only, showed a slightly increased cefoperazone MIC compared to the Δ*epa*_var_ strain, but no increase in the vancomycin MIC was seen. The host adaptation potential of the mutant, complemented, and wild-type strains was analyzed in *in vivo* assays. We compared the levels of virulence of the isogenic strains at two doses of infection in the Galleria mellonella insect model (Fig. S3). At 2 × 10^6^ CFU, a significant difference was observed between the complemented and deleted strains, which showed virulence attenuation in the absence of the *epa*_var_ region and a tendency toward attenuation in comparisons of the deleted and wild-type strains. No difference between the strains was observed at 8 × 10^5^ CFU. The wild-type, Δ*epa*_var_, and complemented strains were also tested in mice to evaluate their ability to colonize the gastrointestinal tract after clindamycin treatment. As observed previously, the wild-type strain transiently colonized the gastrointestinal tract, reaching a median level of 3.2 × 10^8^ CFU/g 1 day after oral inoculation (Fig. 6). The same level of transient colonization was observed with the complemented strain. Conversely, the Δ*epa*_var_ strain was significantly impaired in intestinal colonization. The loss of the *epa*_var_ region reduced the level of day 1 colonization by 1,000-fold (median value of 1.4 × 10^5^ CFU/g). The absence of the *epa*_var_ region also affected persistence, as the deletion mutant strain was below the detection level 11 days after inoculation for 13 of 23 mice. In contrast, the mice inoculated with the wild-type and complemented strains were all persistently colonized, confirming that the *epa* variable region is important for intestinal persistence.

**TABLE 3:** Antibiotic MICs for E. faecalis strains

Strain	Characteristic	MIC (μg/ml)			
		Daptomycin	Vancomycin	Cefoperazone	Lincomycin
VE18379	Wild type	4	32	64	16
VE18385	Δ*epaX*	2	32	128	16
VE18395	Δ*epa*_var_	2	4	64	16
VE18930	Cpl*epa*_var_	4	32	64	1,024^*a*^

a Result represents resistance due to the *ery* resistance provided by the complementation plasmid.

10.1128/mSphere.00310-19.2 FIG S2 Comparison of static growth levels of E. faecalis wild-type (VE18379) and Δ*epa* variable region (VE18395) strains. Download FIG S2, PDF file, 0.02 MB. Copyright © 2019 Furlan et al. 2019 Furlan et al. This content is distributed under the terms of the Creative Commons Attribution 4.0 International license.

10.1128/mSphere.00310-19.3 FIG S3 Survival of Galleria mellonella after injection by E. faecalis wild-type (VE18922), Δ*epa* variable region (VE18927), and complemented *epa* variable region (VE18930) strains. Download FIG S3, PDF file, 0.06 MB. Copyright © 2019 Furlan et al. 2019 Furlan et al. This content is distributed under the terms of the Creative Commons Attribution 4.0 International license.

## DISCUSSION

Here, we report on E. faecalis V583-derived strain VE18379, which showed restored fitness compared to the corresponding VE14089 parental strain, and provide a genetic system to investigate the influence of the specific *epa*_var_ region on host adaptation independently of other genetic variation between strains.

Several loci were identified as linked with fitness during construction of VE18379. This work revealed a role for the transcriptional elongation factor GreA (EF2914) in growth fitness. Yuzenkova et al. (39) demonstrated the important role of GreA of Streptococcus pneumoniae in the regulation of transcription elongation by the resolution of elongation pauses. In E. faecalis OG1RF, GreA is induced in response to copper exposure (36). However, this induction may be indirect and may occur in response to a secondary stress generated by exposure to copper (i.e., oxidative stress). Similarly, global proteome patterns of E. faecalis V583 examined after a pulsed light treatment revealed a level of overproduction of GreA (40) that was consistent with an indirect role of GreA in a general stress response to environmental perturbations. Notably, four of the five mutations of the restored VE18379 strain appeared before *greA* repair, and no SNPs were detected after subsequent deletion of the *epa*_var_ region, suggesting that repair of *greA* lowers the overall mutation rate. Systematic sequencing of future mutants using VE18379 will help to quantify results indicating that repair of GreA stabilized the mutation rate. The recovery of growth rate and cell density after the repair of the *ef2678* gene encoding Spx reveals a role of this protein in the growth fitness of E. faecalis. Spx is a conserved major global stress regulator in low-GC Gram-positive bacteria and was first identified as a suppressor of ClpP and ClpX phenotypes in Bacillus subtilis (41). In E. faecalis OG1RF, inactivation of the *spx* gene impairs growth under aerobic conditions whereas it does not do so under anaerobic conditions (35). That finding mirrors our own observations, where impaired growth was detected only under aerobic conditions. In strain OG1RF, deletion of *spx* resulted in a higher sensitivity to oxidative stress and to *in vitro* macrophage killing using cell line J774. Likewise, a role of Spx in colonization of the peritoneum and spleen in a mouse model of foreign body-associated peritonitis was also reported previously (35). Thus, fitness recovery upon *spx* restoration is in line with the role of Spx in host adaptation of V583 reference-derivative strain VE18379.

During construction of the final VE18379 strain, we noticed improved growth rates after excision of the integrated pTEF1 plasmid sequence that suggested a biological impact of pTEF1 insertion through the activity of pTEF1-carrying genes and/or by polar effects on the surrounding genes. A putative sucrose assimilation operon, *efa0069*-*efa0070*, is present within the integrated region; however, the role of this operon and its potential metabolic advantage remain to be determined. Contiguous to this operon, genes *efA0072* and *efA0071* encode products corresponding to PemI/PemK family proteins that are homologous to the type II toxin-antitoxin system MazE/MazF. Toxin-antitoxin systems are known to be implicated in plasmid inheritance by mediating postsegregation killing (42). In this system, the toxin causes growth inhibition and eventual death of the bacteria but is usually inhibited with its cognate antitoxin (43). The presence of PemI/PemK on the pTEF1 integrated fragment could result in part in a growth disadvantage for E. faecalis VE14089. Insertion of the plasmid sequence was located between two divergent genes, *ef3209* and *ef3210*, each of which is the first gene of its own operon. EF3209 is a predicted ABC transporter ATP-binding protein within an ABC transporter (EF3209-EF3208), and EF3210 is a putative subunit IIA system transporter in a phosphotransferase (PTS) system (EF3210-EF3213), with a sugar specificity for mannose based on KEGG (EC 2.7.1.191). No study has yet revealed a role of these two operons in E. faecalis V583, but transcriptomic analyses revealed very low or undetectable levels of transcripts of genes between *ef3210* and *ef3218* in strain V583 (23, 44). In contrast, analysis of a transcriptome on strain VE14089 (ArrayExpress accession number E-MEXP-3068) revealed that these genes are overexpressed, further suggesting an additional cost for the bacterium of the pTEF1 insertion by overexpression of genes within this region (22). As the artefactual insertion no longer exists in the V583-derived VE18379 strain, it is not influencing the expression of the surrounding genes. In our study, we observed that plasmid pTEF1 or plasmid pTEF2 or both conferred an advantage in intestinal colonization at the time of the peak of antibiotic-induced dysbiosis (D1) but did not confer an advantage in intestinal persistence. This is somewhat in contradiction with the deleterious effect of pTEF2 on intestinal colonization of V583. Using an *in vitro* assay to estimate the competitive growth of E. faecalis V583 in the human gut microbiota, Gilmore et al. (20) reported that V583 was actively killed by endogenous E. faecalis and that the killing was mediated by an heptapeptide pheromone produced by endogenous E. faecalis and was dependent on the presence of plasmid pTEF2. This discrepancy may be explained by the conditions employed for the experiments, as we worked with a mouse model using clindamycin to provide the conditions for an overgrowth of enterococci, as was observed in human clinical cases.

Genes within the *epa* locus have been reported to play a role in several processes such as biofilm formation, adhesion to intestinal mucus and translocation of epithelial cells, resistance to phagocytosis, virulence in infection models, and antibiotic resistance (8, 10, 13, 45). We have shown previously that Epa is a key player in colonization of the intestine by E. faecalis (10). Specifically, we proposed a role for the predicted glycosyl transferase EpaX and, more generally, for the variable region in the decoration of Epa rhamnan backbone putatively coded by the conserved *epa* region. EpaX is involved in cell wall integrity and resistance to bile salts, and a related glycosyltransferase, EpaOX of E. faecalis OG1RF, contributed to biofilm-associated antibiotic resistance (8, 9). The important genetic diversity between E. faecalis strains, occurring independently of the variation of the *epa* locus, may be a confounding factor in assessing a specific role of Epa decorations in host adaptation. Construction of isogenic strains by switching of the *epa*_var_ regions could provide a way to circumvent this confounding diversity, as exemplified previously with capsular serotype-switched strains of Streptococcus pneumoniae (46). This work shows that the complete *epa*_var_ region is dispensable under laboratory growth conditions and confirms a key role of *epa*_var_ in host adaptation, in resistance to biliary salts and detergents and to antibiotics, and in the cell morphology. The Δ*epa*_var_ strain recapitulates phenotypes observed with other single-gene knockouts Δ*epaX* or of genes Δ*epaB*, Δ*epaI*, and Δ*epaR* in the conserved region of *epa*. Our results obtained with daptomycin support the idea of a modification in the physicochemical properties of the cell surface (charge and/or hydrophobicity) due to the Epa structure that modifies resistance. In particular, the absence of the Epa variable region confers higher sensitivity to daptomycin, in line with previous reports (8, 9). Daptomycin is a cyclic lipopeptide antibiotic used to treat infections caused by staphylococci and vancomycin-resistant enterococci (47). The mode of action of daptomycin in S. aureus is that of depolarization of the bacterial membrane and is linked to increased MprF activity of phosphatidylglycerol lysinylation, modifying physicochemical properties of the membrane (48). In contrast to the results seen in studies of S. aureus, the daptomycin resistance of E. faecalis 12030 is not linked to mutation in MprF (49) but involves modification of physicochemical properties by cardiolipin synthase mutation (34), also modifying charge interaction of the bacterial surface. Interestingly, we also established a role of Epa decoration in resistance to vancomycin and to cefoperazone, two antibiotics targeting peptidoglycan synthesis, suggesting a complex form of interplay between the peptidoglycan and Epa decoration chains. Very recently, loss of Epa was shown to be associated with increased resistance to ceftriaxone (another cephalopsporin) mediated by an unknown mechanism (50, 51). Of note, Hoff et al. were the first researchers to observe increased resistance to ceftriaxone of a conditional Δ*epaE* mutant (52). The location and mechanisms of attachment of Epa to the cell wall are not known but have been predicted to be buried within the cell envelope and linked to the peptidoglycan (53). Recently, Smith et al. (54) showed that Epa decoration is required for the peptidoglycan cross-linking and that its defect may impair cell envelope integrity. Additional studies are required to determine how Epa decoration functions in resistance to peptidoglycan-targeting antibiotics. Although the consequences of deletion of *epa* genes have been studied, our understanding of Epa biochemical structure, including the specificity of the secondary chains and how it affects enterococcal pathogenesis and antibiotic resistance, remains incomplete. Our system combining strain VE18395 with plasmid complementation opens avenues to evaluate the links between genetic and biochemical diversity. Construction and investigation of isogenic strains expressing diverse *epa*_var_ regions will help decipher the role of specific moieties of Epa in host adaptation and in resistance to antibiotics. More generally, VE18395 provides a tractable genetic platform for this important opportunistic human pathogen.

## MATERIALS AND METHODS

### Bacterial strains and culture conditions.

Enterococcus faecalis, Escherichia coli, and Lactococcus lactis strains used and constructed in this study are listed in Table S3 in the supplemental material. E. faecalis strains were grown in brain heart infusion (BHI) medium or M17 medium supplemented with 0.5% glucose (GM17) at 37°C. L. lactis cells were grown on GM17 at 30°C. E. coli strains were grown at 37°C in LB medium with shaking. Growth characterization of E. faecalis strains was performed in GM17 broth in under static and agitation conditions. Briefly, 10^6^ bacteria from an exponential-growth-phase culture were inoculated in a volume of 40 ml in plastic tubes (Sarstedt) (50 ml) and were incubated at 37°C. Growth was monitored as the optical density at 600 nm (OD_600_) every 60 min over 10 h. Inocula of E. faecalis for Galleria mellonella and for mouse experiments were prepared as dried frozen pellets as previously described (10, 22). Erythromycin was used at concentrations of 100 μg/ml for E. faecalis, 150 μg/ml for E. coli, and 3 μg/ml for L. lactis for selection of pGh9 and derivatives and at a at concentration of 3 μg/ml for E. faecalis for pIL252 and derivatives.

10.1128/mSphere.00310-19.8 TABLE S3 Bacterial strains and plasmids used in this study. Download Table S3, DOCX file, 0.02 MB. Copyright © 2019 Furlan et al. 2019 Furlan et al. This content is distributed under the terms of the Creative Commons Attribution 4.0 International license.

### Genome resequencing and identification of polymorphisms.

The VE14089 genome was sequenced on a Life Technologies 5500XL NGS system at the MetaQuant platform, INRA Jouy-en-Josas (www.mgps.eu). Mapping of the sequencing reads to the E. faecalis VE14089 reference sequence was performed along with SNP analysis using Lifescope v2.0 software (Life Technologies). To identify variants (insertion, deletions, duplications), mapping data were compared to reference or parental strains by analysis using aligned BAM files in Tablet software, a graphical viewer for next-generation sequence assemblies and alignments (55). Variants identified between VE14089 and V583 (NCBI accession number AE016830) were confirmed by targeted PCR of the affected open reading frame (ORF) on VE14089 and VE14002 DNA (Table S4) followed by sequencing at GATC Biotech (France). Genomic DNA of E. faecalis VE18379 and VE18395 was sequenced on Illumina instruments at GATC Biotech (Constance, Germany), and confirmation of SNPs was performed as described for strain VE14089.

10.1128/mSphere.00310-19.9 TABLE S4 Primers used in this study. Download Table S4, DOCX file, 0.02 MB. Copyright © 2019 Furlan et al. 2019 Furlan et al. This content is distributed under the terms of the Creative Commons Attribution 4.0 International license.

### Construction of E. faecalis strain VE18379 deleted of PTEF1 fragment and with sequential repair of VE14002 genes *ef0295*, *ef2914*, *ef0172*, and *ef2678*.

Construction of strain VE18379 deleted of the pTEF1 fragment and sequential repair of VE14002 genes *ef0295*, *ef2914*, *ef0172*, and *ef2678* were performed through double crossing-over as described previously (56). The regions of interest were subjected to PCR amplification from VE14002 chromosomal DNA. PCR amplifications were made with the primers listed in Table S4. Plasmids obtained in this study are listed in Table S3. Sequencing of the deletion sites was done to confirm the excision of the pTEF1 fragment and the repair of VE14002 wild-type genes using primers detailed in Table S4.

### Galleria mellonella and mouse models to evaluate enterococcal host adaptation.

Galleria mellonella virulence assay was performed as described previously (22). Mouse experiments were carried out in accordance with the European guidelines for the care and use of laboratory animals (Directive 2010/63/UE). The studies received ethical approval from the local ethics committee (COMETHEA) and from the French Ministry of Higher Education and Research (no. 00680.01) for the period 2015 to 2018. The animal facility was accredited by the Direction des Services Vétérinaires (reference A78-187). Mouse experiments were performed in this study as described previously (10). To assess persistence and the ability of E. faecalis to reemerge, subcutaneous clindamycin injections were readministered daily for 3 days at days 8, 9, and 10 or at days 11, 12, and 13. To monitor the inoculated strain of E. faecalis in feces, dilutions of stool samples were plated onto BEA (bile esculin azide agar) supplemented with vancomycin at 6 μg/ml. Comparisons of E. faecalis growth kinetics at each time point were performed by Mann-Whitney test (GraphPad Prism).

### Construction of E. faecalis strain VE18395 deleted of the variable region of *epa* and construction of a plasmid for complementation of the large deletion.

To optimize the genetic tractability of E. faecalis VE18379, we compared the transformation efficiencies of plasmid pGh9 propagated in E. coli JM101 (VE14037) and E. coli GM1674 (VE18916), the latter deleted for the *dam* and *dcm* genes, and in L. lactis MG1363 (see Fig. S4 in the supplemental material). A markerless deletion of the *epa*_var_ region (16,832 bp) from EF2176 to EF2164 was constructed by double homologous recombination using pVE14383, a pGh9-derivative plasmid (57). Two DNA fragments flanking the 5′ and 3′ ends of the *epa*_var_ region gene were amplified by PCR from VE14002 chromosomal DNA with primer pairs OEF823-OEF824 and OEF825–OEF826 (Table S4), respectively. The two PCR products were fused by PCR using the external primers and cloned in pGh9, yielding plasmid pVE14383, using Lactococcus lactis MG1363 *repA* as host. The resulting plasmid was then introduced into E. faecalis VE18379. A markerless in-frame deletion mutant was selected, and the deletion of the variable region was confirmed by sequencing of the chromosomal locus. To complement the Δ*epa* variable region in *trans*, the 16.8-kb *epa*_var_ region was amplified with overlapping primers in 3 fragments as described for Fig. S5 and cloned into low-copy-number Gram-positive plasmid pIL252 (58). Assembly of the four DNA molecules was performed as described previously by Gibson et al. (59) by the single-reaction method using the combined enzymatic activities of a 5′ exonuclease, a DNA polymerase, and a DNA ligase. The resulting pVE14388 plasmid was electroporated into Δ*epa*_var_ strain VE18395 to obtain complemented strain Δ*epa*_var_:pVE14388 (Cpl*epa*_var_). Sequential PCRs and a total of 34 sequencing reactions using primers detailed in Table S4 were performed to verify the sequence of plasmid pVE14388. Control strain Δ*epa*_var_:pIL252, containing an empty plasmid, was also constructed. The Δ*epaX* and complemented strains derived from VE18379 were similarly constructed as described previously in reference 10.

10.1128/mSphere.00310-19.4 FIG S4 Electrotransformability of E. faecalis VE18379 strain with plasmids extracted from different host strains, namely, E. coli JM101 (VE14037) (*dam^+^ dcm^+^*), E. coli GM1674 (VE18916) (deleted for the *dam* and *dcm* genes), and L. lactis MG1363. Results are expressed in number of transformants per microgram of plasmid DNA (means ± standard errors of the means [SEM]). The total number of transformants per microgram of plasmid DNA was obtained for two independent experiments. Comparing the efficiencies of transformation (numbers of transformants per microgram of plasmid DNA), we observed that pGh9 extracted from E. coli GM1674 provided ∼200 times more transformants of VE18379 than methylated pGh9 extracted from E. coli JM101. A higher level of transformation efficiency was observed with pGh9, with ∼250 times more transformants when the plasmid was propagated in L. lactis MG1363. Together, these results showed the importance of restriction and modification (R-M) systems in electrotransformability of the plasmid and that the host strain affects the transformation efficiency of E. faecalis (log_2_). Thus, to improve electrotransformability, we further used plasmids propagated in L. lactis MG1363 to increase transformation efficiencies in E. faecalis VE18379. Bacteria possess genome defense mechanisms to block acquisition of mobile genetic elements; among them are the R-M systems. In our study, we demonstrated that unmethylated plasmid propagated in E. coli deleted of R-M systems or plasmid propagated in L. lactis provided improved electrotransformability of E. faecalis VE18379. This showed that either the absence of modification by the R-M systems of E. coli or modification by the R-M systems of L. lactis, a bacterium that is phylogenetically closer to E. faecalis, confers protection against genome defense mechanisms. Download FIG S4, PDF file, 0.07 MB. Copyright © 2019 Furlan et al. 2019 Furlan et al. This content is distributed under the terms of the Creative Commons Attribution 4.0 International license.

10.1128/mSphere.00310-19.5 FIG S5 Cloning strategy applied to construct the plasmid for complementation of the *epa* variable region of the E. faecalis Δ*epa*_var_ strain. Download FIG S5, PDF file, 0.1 MB. Copyright © 2019 Furlan et al. 2019 Furlan et al. This content is distributed under the terms of the Creative Commons Attribution 4.0 International license.

### *In vitro* characterization of E. faecalis δ*epa*_var_ and its derivatives.

To test SDS and biliary salt sensitivities, E. faecalis cells were grown in BHI medium and were collected 1 h after reaching the stationary phase. Cells were washed twice with a 0.9% saline solution. Serial dilutions of E. faecalis from 5 × 10^6^ to 5 CFU were spotted on plates with BHI medium alone and with BHI medium containing 0.5% and 1% concentrations of sodium cholate, 0.02% and 0.03% concentrations of sodium deoxycholate, and 0.005% and 0.01% concentrations of SDS. Growth was compared in three independent experiments. Transmission electron microscopy (TEM) and polysaccharide profile analysis were performed as described previously (10). Antimicrobial susceptibility testing was performed on VE18379 wild-type, *epa* mutant, and complemented strains and on parental strains VE14002 and VE14089 using the disk diffusion method. The antibiotics tested were penicillin G, ampicillin, oxacillin, piperacillin, imipenem, ceftriaxone, cefoperazone, vancomycin, teicoplanin, bacitracin, polymyxin B, colistin, gentamicin, netilmicin, kanamycin, chloramphenicol, tetracycline, tigecycline, erythromycin, spiramycin, lincomycin, nitrofurantoin, ciprofloxacin, clindamycin, quinupristin-dalfopristin, linezolid, trimethoprim plus sulfamethoxazole, norfloxacin, ofloxacin, enrofloxacin, rifampin, fusidic acid, and mupirocin. Results were interpreted according to the guidelines of the EUCAST (The European Committee on Antimicrobial Susceptibility Testing) committee breakpoint tables for interpretation of MICs and zone diameters (version 9.0, 2019; http://www.eucast.org/). The disk diffusion method was also applied with various concentrations of daptomycin (0.5 to 10 μg·ml^−1^) on BHI medium supplemented with CaCl_2_ at 50 mg/ml. MICs for gentamicin, vancomycin, lincomycin, cefoperazone, and netilmicin were determined by a 2-fold dilution method as described previously (60).

### Data availability.

Genome sequences of E. faecalis strains VE14089, VE18379, and VE18395 have been deposited under GenBank accession numbers CP039296, CP039548, and CP039549, respectively.
