# Supplementary material for: Fitness Restoration of a Genetically Tractable Enterococcus faecalis V583 Derivative To Study Decoration-Related Phenotypes of the Enterococcal Polysaccharide Antigen
Source: mSphere. 2019 Jul 10;4(4):e00310-19. doi: 10.1128/mSphere.00310-19 (PMC6620374; doi:10.1128/mSphere.00310-19)
Supplement: TABLE S1 [file mSphere.00310-19-st001.docx]

Table S1

| Reference position^a^ | Variation type | Variation | Annotation | Predicted function |
| --- | --- | --- | --- | --- |
| 132143 | Insertion | - → G | IR *ef0126-0127* | - |
| 179987 | Deletion | A → - | EF0183 | Hypothetical protein |
| 191992 | SNP | T → A | EF0199 | Ribosomal protein S7 |
| 229297 | Deletion | C → - | IR *ef0252-0253* | - |
| 611688 | SNP | G → T | EF0660 | MATE efflux family protein |
| 872056 | SNP | C → A | EF0906 | Hypothetical protein |
| 921232 | SNP | A → G | EF0958 | PTS system, IIABC components |
| 931162 | SNP | G → T | IR *ef0967-0968* | - |
| 998111 | SNP | C → T | IR *ef1036-1037* | - |
| 1170952 | Insertion | - → T | EF1205 | Transcriptional regulator |
| 1303060 | Deletion | A → - | IR *ef1331-1332* | - |
| 1474120 | SNP | C → A | EF1519 | Cation-transporting ATPase, E1-E2 family |
| 1534568 | Deletion | A → - | EF1581 | Transketolase |
| 1571355 | SNP | C → T | EF1614 | DNA topoisomerase IV, A subunit |
| 1651141 | SNP | T → C | EF1704 | Sensory box histidine kinase |
| 1651146 | SNP | T → C | EF1704 | Sensory box histidine kinase |
| 1917084 | SNP | T → A | EF1979 | ATPase, AAA family |
| 2059477 | Insertion | -- → AA | EF2155 | Phosphoglucomutase family protein |
| 2117617 | Insertion | - → C | EF2204 | Aminopeptidase |
| 2232992 | Deletion | T → - | EF2308 | Hypothetical protein |
| 2234179 | Insertion | - → G | IR *ef2311-2312* | - |
| 2234474 | SNP | C → G | EF2312 | DNA topoisomerase III |
| 2309182 | SNP | C → A | IR *ef2380-2381* | - |
| 2323031 | Deletion | C → - | EF2399 | Acetyltransferase |
| 2325873 | Insertion | - → G | EF2405 | Hypothetical protein |
| 2452948 | Insertion | - → C | IR *ef2528-2529* | - |
| 2452957 | Insertion | - → C | IR *ef2528-2529* | - |
| 2570780 | SNP | T → C | IR *ef2658-2659* | - |
| 2578163 | SNP | C → G | IR *ef2665-2666* | - |
| 2677895 | Deletion | A → - | EF2772 | Drug resistance transporter |
| 3000492 | Insertion | - → C | EF3124 | Polypeptide deformylase |
| 3015992 | SNP | T → A | IR *ef3142-3144* | - |
| 3100859 | Deletion | G → - | EF3231 | Ribosomal protein L13 |
| 3125849 | Insertion | - → C | EF3251 | Hypothetical protein |

^a^Position in *E. faecalis* V583 reference sequence in NCBI (Acc number: AE016830).
