# Supplementary figures and images for: Fitness Restoration of a Genetically Tractable Enterococcus faecalis V583 Derivative To Study Decoration-Related Phenotypes of the Enterococcal Polysaccharide Antigen
Source: mSphere. 2019 Jul 10;4(4):e00310-19. doi: 10.1128/mSphere.00310-19 (PMC6620374; doi:10.1128/mSphere.00310-19)

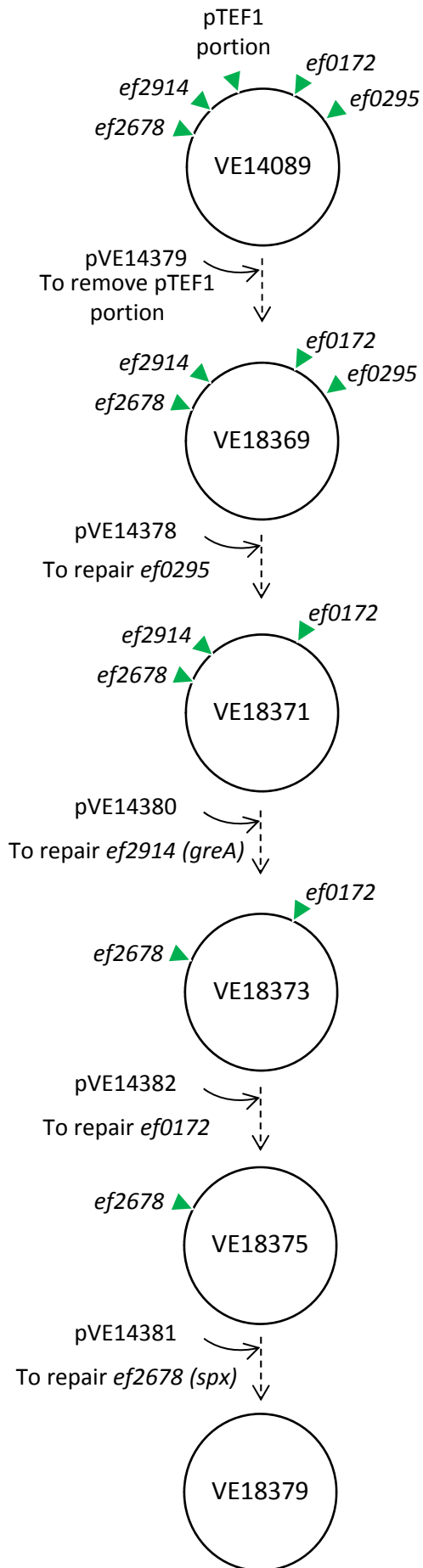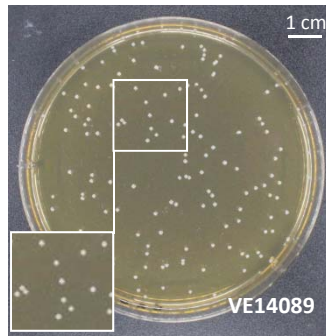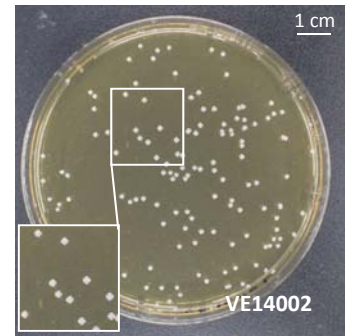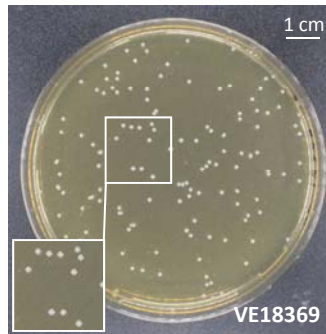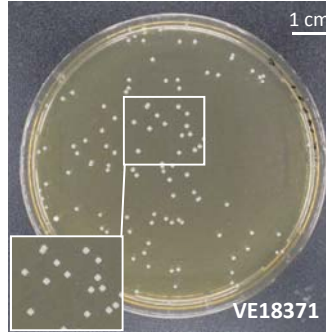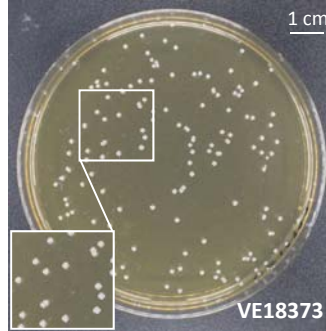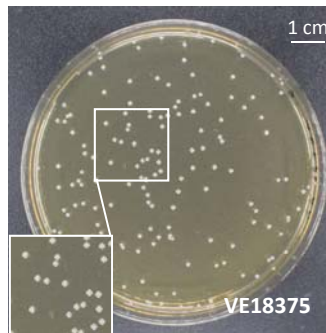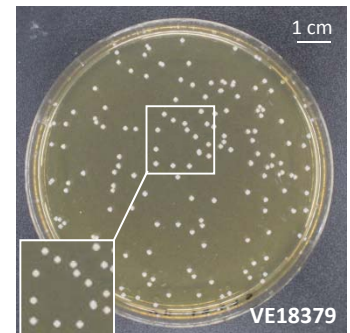

Supplement: FIG S1 [file mSphere.00310-19-sf001.pdf]

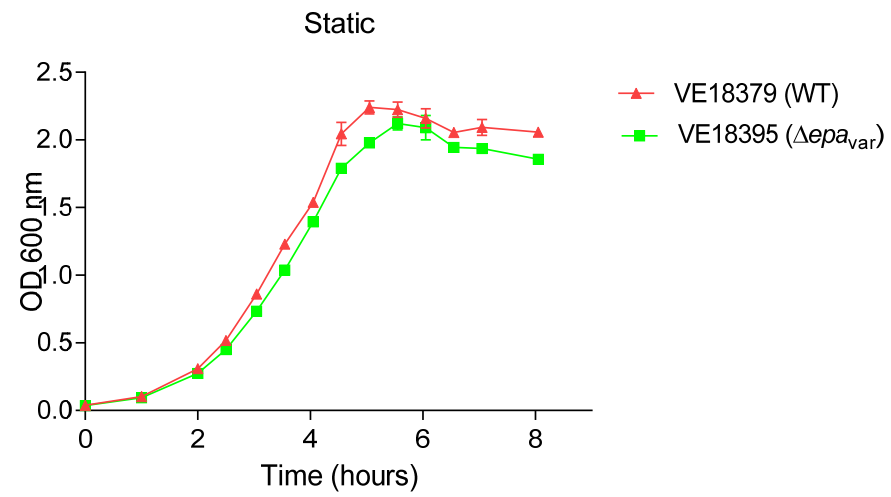

Supplement: FIG S2 [file mSphere.00310-19-sf002.pdf]

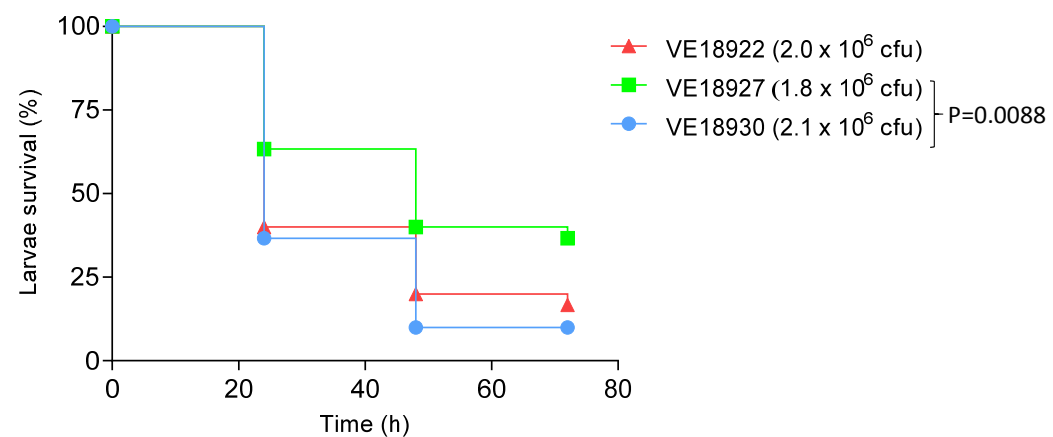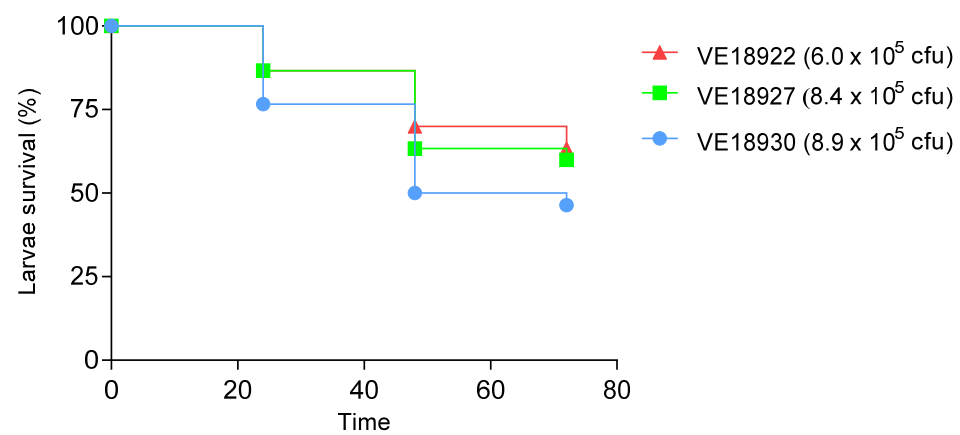

Supplement: FIG S3 [file mSphere.00310-19-sf003.pdf]

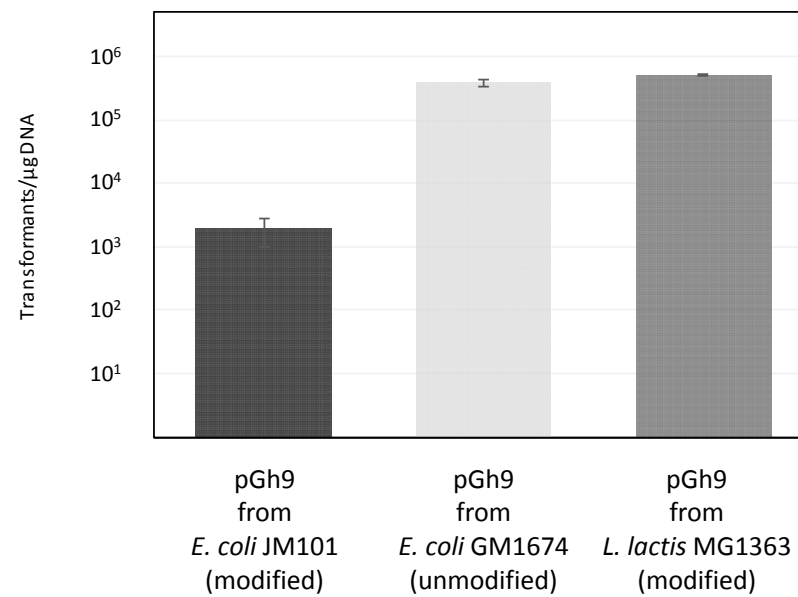

Supplement: FIG S4 [file mSphere.00310-19-sf004.pdf]

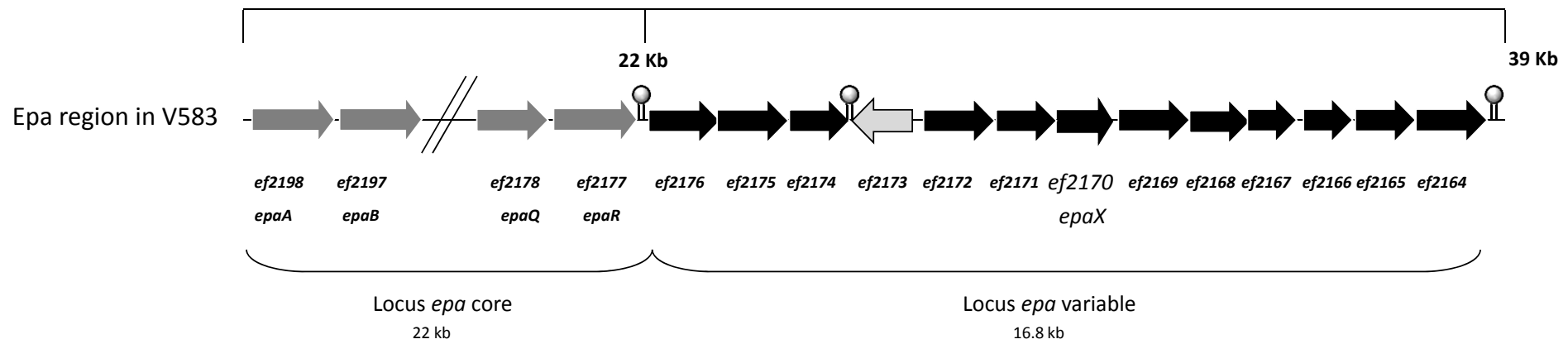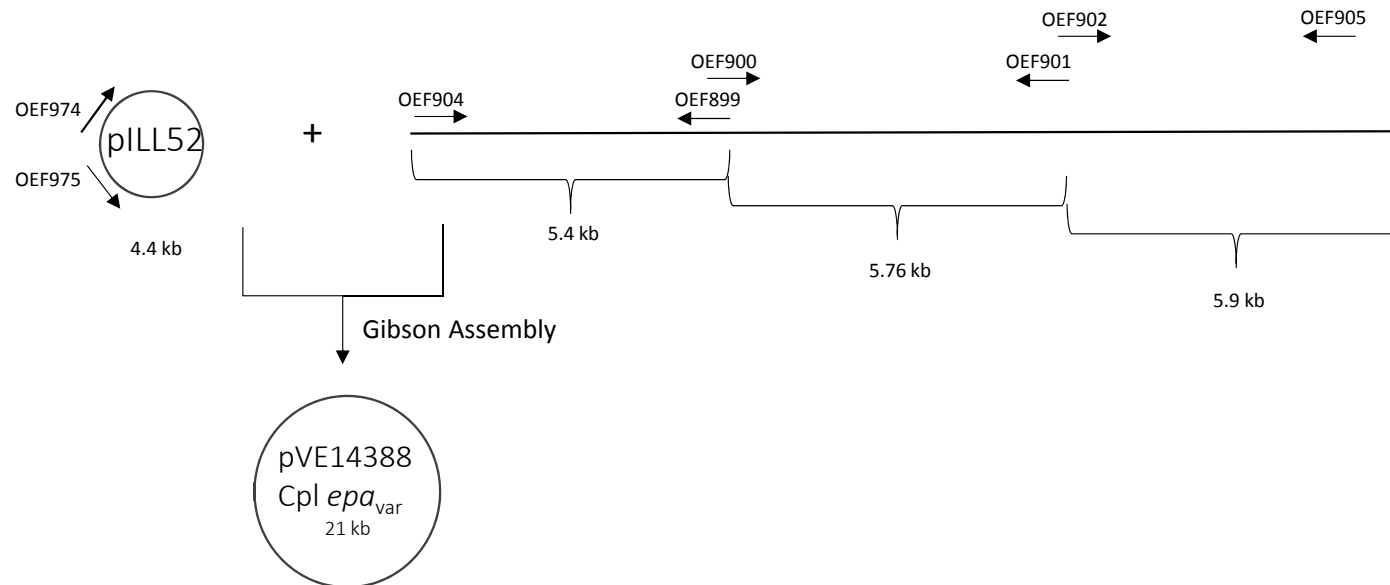

Supplement: FIG S5 [file mSphere.00310-19-sf005.pdf]
