# Supplementary material for: Fitness Restoration of a Genetically Tractable Enterococcus faecalis V583 Derivative To Study Decoration-Related Phenotypes of the Enterococcal Polysaccharide Antigen
Source: mSphere. 2019 Jul 10;4(4):e00310-19. doi: 10.1128/mSphere.00310-19 (PMC6620374; doi:10.1128/mSphere.00310-19)
Supplement: TABLE S2 [file mSphere.00310-19-st002.docx]

Table S2

|  | Static |  |  | Agitation |  |
| --- | --- | --- | --- | --- | --- |
|  | Doubling time (h)  (mean±SEM) | Cell density^a^  (OD±SEM) | CFU counts^b^ | Doubling time (h)  (mean±SEM) | Cell density^a^  (OD±SEM) |
| VE14089 | 1.64±0.01 | 1.75±0.03 | 0.8×10^8^ | 1.32±0.08 | 2.37±0.01 |
| VE18369 | 1.27±0.1 | 2.0±0.1 | 1.4×10^8^ | 1.27±0.2 | 2.46±0.0 |
| VE18371 | 1.12±0.07 | 1.99±0.0 | 1.5×10^8^ | 1.2±0.04 | 2.48±0.04 |
| VE18373 | 1.18±0.02 | 2.18±0.02 | 2.0×10^8^ | 0.95±0.02 | 2.66±0.08 |
| VE18375 | 1.19±0.02 | 2.17±0.01 | 2.2×10^8^ | 0.99±0.06 | 2.68±0.07 |
| VE18379 | 0.99±0.02 | 2.33±0.01 | 2.3×10^8^ | 0.83±0.04 | 3.2±0.18 |
| VE14002 | 0.99±0.01 | 2.39±0.06 | 2.2×10^8^ | 0.76±0.01 | 3.37±0.1 |

^a^OD-600nm cell density one hour after the entrance in stationary phase

^b^CFU counts in exponential phase
