# Supplementary material for: Fitness Restoration of a Genetically Tractable Enterococcus faecalis V583 Derivative To Study Decoration-Related Phenotypes of the Enterococcal Polysaccharide Antigen
Source: mSphere. 2019 Jul 10;4(4):e00310-19. doi: 10.1128/mSphere.00310-19 (PMC6620374; doi:10.1128/mSphere.00310-19)
Supplement: TABLE S3 [file mSphere.00310-19-st003.docx]

| Table S3 | | |  |  |
| --- | --- | --- | --- | --- |
| Species/Plasmid | |  | |  |
| Strains (Acc number) | | Origin and/or characteristics | | Reference |
| *Enterococcus faecalis* | |  | |  |
| VE14002 | | V583 | | (18, 32) |
| VE14089 (CP039296) | | V583 plasmid cured derivative | | (22) |
| VE18369 | | VE14089 with excised pTEF1 portion | | This study |
| VE18371 | | VE18369 with *ef0295* of V583 | | This study |
| VE18373 | | VE18371 with *ef2914* (*greA*) of V583 | | This study |
| VE18375 | | VE18373 with *ef0172* of V583 | | This study |
| VE18379 (CP039548) | | VE18375 with *ef2678* (*spx*) of V583 | | This study |
| VE18385 | | VE18379 Δ*epaX* | | This study |
| VE18387 | | VE18385 complemented *epaX* | | This study |
| VE18395 (CP039549) | | VE18379 Δ*epa*_var_ region | | This study |
| VE18930 | | VE18395 complemented *epa*_var_ region | | This study |
| VE18922 | | VE18379 pIL252 | | This study |
| VE18927 | | VE18395 pIL252 | | This study |
| VE18940 | | VE18379 pVE14176 | | This study |
| VE18386 | | VE18385 pVE14176 | | This study |
| *Lactococcus lactis* | |  | |  |
| LL108 | | MG1363 carrying *repA* gene | | K. Leenhouts *et al*. Mol Gen Genet 253:217-224, 1996 |
| VE18380 | | MG1363 *repA*+pGh9 | | This study |
| VE18356 | | VE18360 + pVE14378, Erm^r^ | | This study |
| VE18361 | | VE18360 + pVE14379, Erm^r^ | | This study |
| VE18362 | | VE18360 + pVE14380, Erm^r^ | | This study |
| VE18363 | | VE18360 + pVE14381, Erm^r^ | | This study |
| VE18366 | | VE18360 + pVE14382, Erm^r^ | | This study |
| *Escherichia coli* | |  | |  |
| VE14037 | | JM101 *repA*+pGh9 | | S. Nouaille (unpublished) |
| VE18916 | | GM1674 *repA* (VE14188)+pGh9 | | This study |
| Plasmids | |  | |  |
| pGh9 | | Erm^r^, *repA* TS | | (56) |
| pIL252 | | Erm^r^ | | (57) |
| pVE14176 | | Erm^r^ | | R. Dumoulin *et al*. J Bacteriol 195:3073-3083, 2013 |
| pVE14297 | | Erm^r^ to complement Δ*epaX* | | (10) |
| pVE14379 | | Erm^r^, pGh9 with ΔpTEF1 deletion (PCR OEF766+OEF767) (*ApaI/ClaI*) | | This study |
| pVE14378 | | Erm^r^, pGh9 with *ef0295* of V583 (PCR OEF763+OEF764) (*ApaI*/*PstI*) | | This study |
| pVE14381 | | Erm^r^, pGh9 with *ef2678* of V583 (PCR OEF770+OEF771) (*ApaI*/*PstI*) | | This study |
| pVE14380 | | Erm^r^, pGh9 with *ef2914* of V583 (PCR OEF774+OEF775) (*ApaI*/*PstI*) | | This study |
| pVE14382 | | Erm^r^ pGh9 with *ef0172* of V583 (PCR OEF782+OEF783) (*ApaI*/*PstI*) | | This study |
| pVE14383 | | Erm^r^ pGh9 with flanking genes of *epa* variable region (fusion of PCR products of OEF823-OEF824 and OEF825-OEF826) (*ApaI*/*XmaI*) | | This study |
| pVE14388 | | Erm^r^ pIL252 with 16.8 kb of *epa* variable region of V583 obtained by Gibson assembly as detailed in Figure S5 | | This study |
|  |  | | |  |
|  |  | | |  |
