# Supplementary material for: Fitness Restoration of a Genetically Tractable Enterococcus faecalis V583 Derivative To Study Decoration-Related Phenotypes of the Enterococcal Polysaccharide Antigen
Source: mSphere. 2019 Jul 10;4(4):e00310-19. doi: 10.1128/mSphere.00310-19 (PMC6620374; doi:10.1128/mSphere.00310-19)
Supplement: TABLE S4 [file mSphere.00310-19-st004.docx]

| Table S4 | | |  |
| --- | --- | --- | --- |
| Name | Target | Sequence | Reference^a^ |
| OEF701 | EF0172 | AATGCAAAAGGAGACGTTGG |  |
| OEF702 | EF0172 | AATCGTCCACTATCTCGTG |  |
| OEF703 | EF0295 | ACAAAGAAATGGTAGAGGAG |  |
| OEF704 | EF0295 | CAAATCAAAGCCAGCATTAC |  |
| OEF707 | EF0573 | CCACAAATTACTAGTGCAG |  |
| OEF708 | EF0573 | TCTCCTTTAGCATCTACTTG |  |
| OEF709 | EF1007 | AGTCTGTGTATGAAGTACCC |  |
| OEF710 | EF1007 | TAAAGCGCTAGGAGTTCTTC |  |
| OEF711 | EF1021 | TTTTAATCAGGAACCCACGG |  |
| OEF712 | EF1021 | TTCTGCTTTCCCTTCTGACG |  |
| OEF721 | EF1976 | TAGAACTGTTTCGCCACCAC |  |
| OEF722 | EF1976 | AGAGGTGAGAACATGAAGTG |  |
| OEF713 | EF2060 | TCTGCATAATTTCGAGCACG |  |
| OEF714 | EF2060 | TTGGCGAATGAGTACACTAC |  |
| OEF717 | EF2678 | CCTGCCATTAATTGTGCTTG |  |
| OEF718 | EF2678 | TTCGCGAAAAGGAGTGATGC |  |
| OEF719 | EF2914 | GTCATAGATTGCGAAAGGAC |  |
| OEF720 | EF2914 | GAACTACGTATCGTTAAGCG |  |
| OEF761 | EF0295 | AAGTAGCCCAATTATCCTAG |  |
| OEF762 | EF0295 | AACACACCAAACGTAGTGG |  |
| OEF763 | EF0295 | TAGGGCCCTTGCAGCGAGTATTGATGG |  |
| OEF764 | EF0295 | GTCTGCAGCAAAATCGTGACAATCAGG |  |
| OEF766 | pTEF1 | TAGGGCCCACTGAATAATCCCAATGTTGC |  |
| OEF767 | pTEF1 | ATAATCGATCATTCGCTACAACTAATAAGG |  |
| OEF768 | pTEF1 | TTAACTCGTCCTCAAATACG |  |
| OEF769 | pTEF1 | GACATCAATCGCATCATCG |  |
| OEF 780 | pTEF1 | ATCTTAACTGTTGAAGCACC |  |
| OEF 781 | pTEF1 | AATCAACGCAACTTCTATCC |  |
| OEF770 | EF2678 | TAGGGCCCTCGTTGCCAATTAACACACG |  |
| OEF771 | EF2678 | GTCTGCAGTCATTCGCTCTAACTCACC |  |
| OEF772 | EF2678 | TAAGGAAGACAAGTCGTCC |  |
| OEF773 | EF2678 | TTGTACAACACGATATCACC |  |
| OEF789 | EF2678 | TTCCTTCATTACATCCGTG |  |
| OEF774 | EF2914 | TAGGGCCCAGGATTATGTACAATTTGC |  |
| OEF775 | EF2914 | GTCTGCAGCAAAGAATTTAACCAATCAGC |  |
| OEF776 | EF2914 | TTAAGCCGATAAAGAGTACG |  |
| OEF777 | EF2914 | CTTAGAAGGCTATTTGTTCC |  |
| OEF782 | EF0172 | TAGGGCCCTTACACCAAAATCTTCTTCGC |  |
| OEF783 | EF0172 | GTCTGCAGCCATTTCATCATTGCCAC |  |
| OEF784 | EF0172 | GGTTGGCTAAAGCTAATACG |  |
| OEF785 | EF0172 | GATAGACAGTTGTTAAACTCG |  |
| OEF791 | IR EF0251-EF0252 | CTATCAGTTTTAACAGGAGG |  |
| OEF792 | IR EF0251-EF0252 | CAGAACTATTTTCAGCTACC |  |
| OEF793 | EF1093 | AATGTCACTGTTCAGTTACC |  |
| OEF794 | EF1093 | CTGTTAATTCTCCCGTTCC |  |
| OEF795 | IR EF1331-EF1332 | GACTAACATAAACGATGAGG |  |
| OEF796 | IR EF1331-EF1332 | TGTTCAACCGAGAAACTGG |  |
| OEF797 | EF0073 | AAAGCTCGGGAATATTTAGC |  |
| OEF798 | EF0073 | TTTGGCGTAGAAACAGAGG |  |
| OEF799 | EF0077 | TAATGTCCAATGGTTCTTGC |  |
| OEF800 | EF0077 | ACTAGGCCACCAATTAACG |  |
| OEF801 | EFtRNAAsp2 | TCATAGTTTTGTTACGGAGG |  |
| OEF802 | EFtRNAAsp2 | CTTCCCCAAGAAATAAATGG |  |
| OEF803 | IR EF0420-EF0421 | CGTCGTTAAAAGCGTTTGG |  |
| OEF804 | IR EF0420-EF0421 | TTTGACGACTGTATGTAACC |  |
| OEF805 | EF1054 | AAGTGGCTCGAACTAAAGG |  |
| OEF806 | EF1054 | CAACTGCTTGGTTACTTGG |  |
| OEF807 | IR EFtRNALys3-EF1451 | TGTTATTCAGTGTGGATTCG |  |
| OEF808 | IR EFtRNALys3-EF1451 | CTTGATACTCATCTATAGCC |  |
| OEF809 | EF1538 | AGGACATGCAGATATCTCC |  |
| OEF810 | EF1538 | CTGAGCTACTAAAGCTTCC |  |
| OEF811 | EF1976 (*prmA*) | AGTTTCTAAAGCTTGCAAGG |  |
| OEF812 | EF1976 (*prmA*) | AGGTGAGAACATGAAGTGG |  |
| OEF813 | EF2405 | GTGGTTACTCCTATCAAGG |  |
| OEF814 | EF2405 | ACGATGGTTATGGTTGAGG |  |
| OEF815 | IR EF2437-EF2438 | TAAATCAGTCAAGCGTTGG |  |
| OEF816 | IR EF2437-EF2438 | TGATACGGTAGAGTTAAACG |  |
| OEF817 | EF2466 | TTCTTGTATCCGTATCTTGC |  |
| OEF818 | EF2466 | AGTTAGAACAAACACAGACG |  |
| OEF819 | IR EF3310-*gidA* | TGCTCGAGAAGTTACATCG |  |
| OEF820 | IR EF3310-*gidA* | TCCAACCAGAAACGATTGC |  |
| OEF849 | EF0838 | TAGCCAAAGAGCAAATTCG |  |
| OEF850 | EF0838 | CAATGTGTTTTTTCATCATGG |  |
| OEF851 | EF2976 | CTAAGCACACCAAATAAACC |  |
| OEF852 | EF2976 | ACGTTACGGTATCATTATCG |  |
| OEF853 | EF2976 | GAGATTGTTTTACCTAAAGG |  |
| OEF854 | EF2976 | TTGTAGAACTTCTGATAAGC |  |
| OEF823 | EF2177 | TAGGGCCCAAGTAATGTTGATGAGAACG |  |
| OEF824 | EF2176 | TCCATGGTTACTGCATGCTTCAACGTTCCTTGTTTCC |  |
| OEF825 | EF2163 | AAAGCCCGGGTTCTTCTTGCCAATTTTGACG |  |
| OEF826 | EF2164 | AGCATGCAGTAACCATGGATCCAGGAATGCGTTAGTTG |  |
| OEF827 | EF2177 | TCTGTTTCTGTGACTTTAGC |  |
| OEF828 | EF2163 | CTTAAAGGTCAGCAATGAGC |  |
| OEF974 | pIL252/EF2176 | AACGGTAAATTCTAATAGCATTCTCCTAACAACAAGACACACAC |  |
| OEF975 | pIL252/EF2164 | CTACATTATTTGAACCCACCTCACAGAGAAGGCGAAAACATTGC |  |
| OEF904 | EF2176 | GAGAATGCTATTAGAATTTACC |  |
| OEF899 | EF2173 | AGCTAACTCAAAAGAAGAAGC |  |
| OEF900 | EF2173 | GTATTCGCTTCTTCTTTTGAG |  |
| OEF901 | EF2168 | AACTCCTAAGTCCTTATCTTC |  |
| OEF902 | EF2168 | GTGGAGAAGATAAGGACTTAG |  |
| OEF905 | EF2164 | GTGAGGTGGGTTCAAATAATG |  |
| OEF879* | pIL252 | GGATTGGATTAGTTCTTGTG |  |
| OEF859* | EF2172 | AATTATCGGTTTTTCCTCTACC |  |
| OEF856* | EF2175 | CTTTATTCTGTTCACAAGTGG |  |
| OEF528* | EF2170 | TCATTCTTCTCCCAAGCTTC |  |
| OEF527* | EF2170 | TGCTCGTAATGCTGGAATTG |  |
| OEF857* | EF2166 | AAATCTCGTCCTCCTAATGC |  |
| OEF858* | EF2167 | ATCTATACGCATCTGAAATGG |  |
| OEF903* | pIL252 | AGTAATTCATTCCTGGTTGC |  |
| OEF869 | IR 2176-2177 | CTTACATAAAATGCAACGAGG |  |
| OEF830 | IR 2176-2177 | TATTTCTGGTCTTTGCAATGC |  |
| OEF980 | EF2175 | AGTAGAAATTCAACAAGTCG |  |
| OEF981 | EF2174 | TTAGTGTGATTGCATCAGG |  |
| OEF855 | EF2174 | TTATGGTTCGTTCCTTCACG |  |
| OEF1016 | EF2174 | TTCTAAACGCTACATCTTGG |  |
| OEF982 | EF2174 | CAGCAATTAAAGGCATTAGG |  |
| OEF983 | EF2174 | GCAAATAAAGTATCTGCAGG |  |
| OEF984 | EF2173 | CTAATGTATCGGTTACCTG |  |
| OEF985 | EF2173 | ACCTAGCATTCGTAGAACC |  |
| OEF986 | IR EF2173-*ispD* | AGTTCGATTAAGGTCTAC |  |
| OEF394 | EF2171 | AGTTATCGGAGATGTCACAG | (10) |
| OEF987 | EF2171 | AAATATGCTGCGTACCAGC |  |
| OEF988 | EF2169 | GTAGAGTCTTTGTATTACG |  |
| OEF399 | EF2169 | AGAACATCCCGAAAATTACC |  |
| OEF989 | EF2169 | TAACTCTGCTAAATTGCTG |  |
| OEF902 | EF2168 | GTGGAGAAGATAAGGACTTAG |  |
| OEF990 | EF2168 | GTAGGAAACAATCAATATTGG |  |
| OEF991 | EF2166 | TTGACGGCCATTGTTTCAG |  |
| OEF992 | EF2166 | GCATTTCGTTGTAATTTAGC |  |
| OEF1017 | EF2165 | TTTCGGTTCATAACCAATGG |  |
| OEF993 | EF2165 | ATATGATGTTCCAACAAGC |  |
| OEF994 | EF2164 | AGCATTAGATTACGGACG |  |
| OEF995 | EF2164 | AACCATTACGTTCATGAAG |  |
| OEF829 | EF2164 | GACTAATTGGTAAAATTGAAGC |  |
| OEF1009 | pIL252 (IR after *repE*) | TAACGAGTGAAAAAGTACTC |  |
| OEF997 | pIL252 (*ermAM*) | GTTTCAAAATGGGTCAATCG |  |
| OEF1006 | pIL252 (*repE*) | CAAAGGGATATTCATTCAGC |  |
| OEF1007 | pIL252 (*repE*) | TCGTTTCGTGCAATAAGAG |  |
| OEF1008 | pIL252 (*repE*) | CTAATCATTCGCTAAACAGC |  |
|  |  |  |  |

Black for pGh9 and pIL252 construction

Red for check DCO and sequencing

Blue for resequencing

*Oligos for sequential PCR on pVE14388

^a^This study unless otherwise stated
